# Evolutionary relationships among bifidobacteria and their hosts and environments

**DOI:** 10.1186/s12864-019-6435-1

**Published:** 2020-01-08

**Authors:** Cynthia I. Rodriguez, Jennifer B. H. Martiny

**Affiliations:** Department of Ecology and Evolutionary Biology, University of California, Irvine, CA 92697 USA

**Keywords:** *Bifidobacterium*, Pan-genome, CAZymes, Host-trait associations

## Abstract

**Background:**

The assembly of animal microbiomes is influenced by multiple environmental factors and host genetics, although the relative importance of these factors remains unclear. Bifidobacteria (genus *Bifidobacterium,* phylum Actinobacteria) are common first colonizers of gut microbiomes in humans and inhabit other mammals, social insects, food, and sewages. In humans, the presence of bifidobacteria in the gut has been correlated with health-promoting benefits. Here, we compared the genome sequences of a subset of the over 400 *Bifidobacterium* strains publicly available to investigate the adaptation of bifidobacteria diversity. We tested 1) whether bifidobacteria show a phylogenetic signal with their isolation sources (hosts and environments) and 2) whether key traits encoded by the bifidobacteria genomes depend on the host or environment from which they were isolated. We analyzed *Bifidobacterium* genomes available in the PATRIC and NCBI repositories and identified the hosts and/or environment from which they were isolated. A multilocus phylogenetic analysis was conducted to compare the genetic relatedness the strains harbored by different hosts and environments. Furthermore, we examined differences in genomic traits and genes related to amino acid biosynthesis and degradation of carbohydrates.

**Results:**

We found that bifidobacteria diversity appears to have evolved with their hosts as strains isolated from the same host were non-randomly associated with their phylogenetic relatedness. Moreover, bifidobacteria isolated from different sources displayed differences in genomic traits such as genome size and accessory gene composition and on particular traits related to amino acid production and degradation of carbohydrates. In contrast, when analyzing diversity within human-derived bifidobacteria, we observed no phylogenetic signal or differences on specific traits (amino acid biosynthesis genes and CAZymes).

**Conclusions:**

Overall, our study shows that bifidobacteria diversity is strongly adapted to specific hosts and environments and that several genomic traits were associated with their isolation sources. However, this signal is not observed in human-derived strains alone. Looking into the genomic signatures of bifidobacteria strains in different environments can give insights into how this bacterial group adapts to their environment and what types of traits are important for these adaptations.

## Background

Bacteria are central to the evolution and ecology of animals influencing their genomes, development, and physiology [1]. The composition of bacterial communities in the animal gut are thought to be shaped by host physiology and diet on daily timescales, but also by host evolutionary history over much longer timescales [2–4]. A major challenge in animal microbiome research is therefore to disentangle the ecological and evolutionary processes underlying the variation in gut communities. One approach to tackling these questions is to focus on a specific bacterial group within the larger gut community [5,6].

A widespread and abundant group of bacteria in mammalian guts is bifidobacteria. Bifidobacteria are gram-positive, anaerobic, saccharolytic bacteria, members of the genus *Bifidobacterium* of the phylum Actinobacteria [7]. Their presence in the gut has been correlated with health-promoting benefits in humans and mouse models including the production of metabolites like vitamins and antioxidants, immune system development, and protection from certain gut diseases such as enterocolitis and acute diarrhea [8]. In newborns, specific species of bifidobacteria are important for degrading human milk oligosaccharides (HMOs) derived from breast milk [9,10]. The fermentation of HMOs promotes the wellness of infants and prevents colonization from potential pathogenic bacteria [11, 12]. Bifidobacteria also excel at degrading and fermenting carbohydrates [13, 14]. This process produces short-chain fatty acids (SCFAs) such as butyrate, acetate, and propionate, which have been linked to reducing the risk of inflammatory diseases, heart disease, type II diabetes, and other adverse conditions such as cancer [15].

Here, we take a comparative genomics approach to investigate the relationship between bifidobacteria diversity and their hosts and environments. Bifidobacteria are ubiquitous inhabitants of the gastrointestinal tract, vagina, and mouth of mammals, including humans and are also present in guts of insects such as bees [16,17]. They have also been found in human blood, breast milk, and sewage [18–20]. The genomic signatures of bifidobacteria strains in different environments can give insights into how this bacterial group adapts to their environment and what types of traits are important for these adaptations. The few studies that have considered the association between bifidobacteria diversity and their hosts and environments have found contradictory results. Some studies observe no relationship between hosts and the type of genes bifidobacteria carry [21,22], while others do [23–25].

We analyzed a subset of the over 400 bifidobacteria genomes publicly available to answer two questions: 1) Do bifidobacteria show a phylogenetic signal with their isolation sources (hosts and environments)? and 2) Do key traits encoded by the bifidobacteria genomes depend on the host or environment from which they were isolated? The term “phylogenetic signal” generally refers to the tendency of related species to resemble one another more than they would resemble a species drawn randomly from the same phylogenetic tree [26,27].

Since most bacterial traits are phylogenetically conserved [28], our first hypothesis was that bifidobacteria are adapted to the hosts (and other environments) from which they are isolated. We predicted that this adaptation would be reflected in the phylogeny of bifidobacteria, despite horizontal gene transfer (HGT) and rapid evolution. Secondly, we hypothesized that bifidobacteria strains would further adapt to their environment through genomic signatures like genome size and overall composition of accessory genes, as well as the composition of particular traits. Genome size is broadly associated with different bacterial lifestyles [29–31], and accessory gene composition can capture horizontally transferred regions of the genome, which are thought to allow for rapid adaptation to a specific environment [32]. We specifically focused on two particular classes of genes: amino acid biosynthesis genes and carbohydrate-active enzymes (CAZymes). The abundance and diversity of amino acid biosynthesis genes may vary as amino acids can be exchanged between different hosts and bacteria [33,34], allowing for the loss or gain of these genes. Bacterial CAZyme profiles are also known to vary by environment, suggesting a mechanism for bacteria to adapt to the local carbohydrate supply [35,36]. Moreover, bifidobacteria are key degraders of carbohydrates in host guts, and we expected that strains might adapt to host diet.

## Results

### Phylogenetic relationships between bifidobacteria strains and isolation sources

**Fig. 1:**
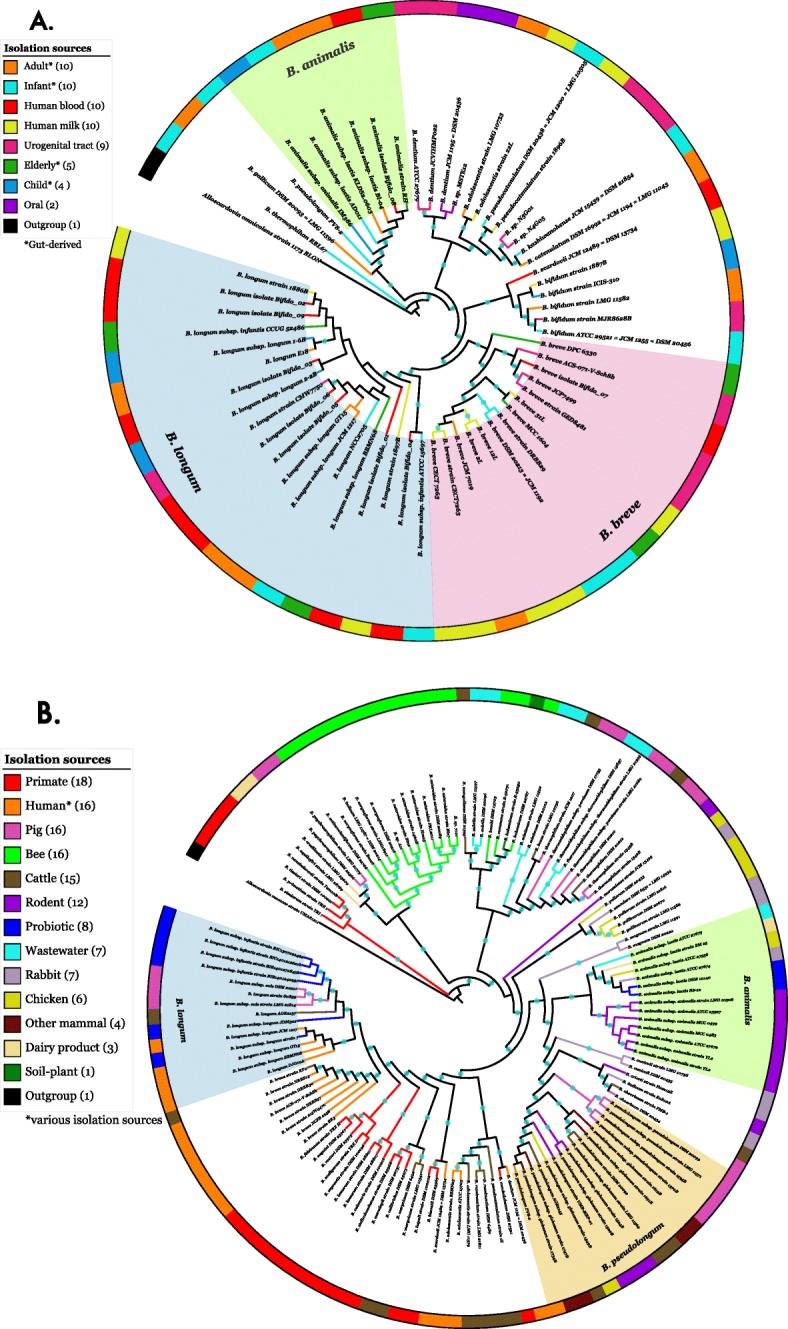
Cladograms of bifidobacteria strains harbored by A) human hosts and B) multiple hosts and environments. **1A**|Phylogenetic relationship of human-derived *Bifidobacterium* strains based on 107 marker genes (*n* = 60 + outgroup). The prominent species, *B. longum* (31.7%), *B. breve* (23.3%), and *B. animalis* (10%), are shaded in different colors. **1B**|Phylogenetic relationship of *Bifidobacterium* strains harbored by multiple hosts based on 107 marker genes (*n* = 129 + outgroup). The prominent species, *B. pseudolongum* (12.4%), *B. longum* (10.9%) and *B. animalis* (10.1%), are shaded in different colors. For both cladograms, the outermost ring represents the different isolation sources. Bootstrap values higher than 70% are represented with blue circles. Strains from the *Alloscardovia* genus were used as outgroups for both phylogenetic trees (accession numbers JWAI01000000 and NEKB01000000). Note that the “child” category refers to ages 2 through 6 years old while “infant” is younger. The “mammal” category indicates a mammal with only 1 sample size, including giraffe (n = 1), hippopotamus (n = 1), llama (n = 1), and wallaby (n = 1). Also, the “primate” category indicates non-human primates, and “probiotic” had an original, unknown isolation source that may overlap with the other categories

To investigate the phylogenetic relationships between bifidobacteria strains isolated from different environments and hosts, two phylogenetic trees were constructed based on 107 concatenated core genes. These trees included one with 60 human-derived strains (Fig. 1a) and one with 129 strains from different environments and hosts (Fig. 1b). In both trees, members of the same taxonomic species clustered closely, and the phylogenetic structure of the trees was similar to previous reports based on 16S rRNA sequences and based on various core genes [16,24,37–39]. For instance, *B. breve* and *B. longum* strains were found to be closely related as well as *B. bifidum* and *B. scardovii*. One difference was that the *B. asteroides* phylogroup has been previously shown to be positioned in the deepest branches of the bifidobacteria lineage [16,24,40]; however, in our human-derived strains phylogenetic tree the deepest branch corresponded to a member of the *B. thermophilum* species; perhaps, this is due to the fact that we did not have a representative of the *B. asteroides* phylogroup to include in the human-derived tree. In the larger tree, the deepest branches corresponded to strains from the *B. simiarum, B. primatium, B. vansinderenii*, and *B. tissieri* species.

The strains isolated from a variety of human stages and body locations showed no phylogenetic signal (ANOSIM: R = 0.022, *p* > 0.05). For example, strains isolated from infants were not more genetically similar to one another than those isolated from adults (Fig. 1a). Similarly, strains isolated from the blood were not more genetically similar to one another than those found in milk or in the urogenital tract.

By contrast, when comparing across multiple host species and environments, the habitat from which the strains were isolated was strongly associated with the bacteria’s phylogenetic distribution (Fig. 1b**;** ANOSIM: R = 0.420, *p* < 0.001). For instance, bee, primate, and rodent derived strains are tightly clustered in the phylogenetic tree within their categories (Fig. 1b). These broader evolutionary patterns seem particularly robust for strains isolated from the orders Artiodactyla (pig and cattle-derived strains), Hymenoptera (bee-derived strains), and Primates (human and non-human primate-derived strains) as they clustered mostly within the same branches (Fig. 1b).

### Genomic features and content among isolation sources

#### Genome size analysis

**Fig. 2:**
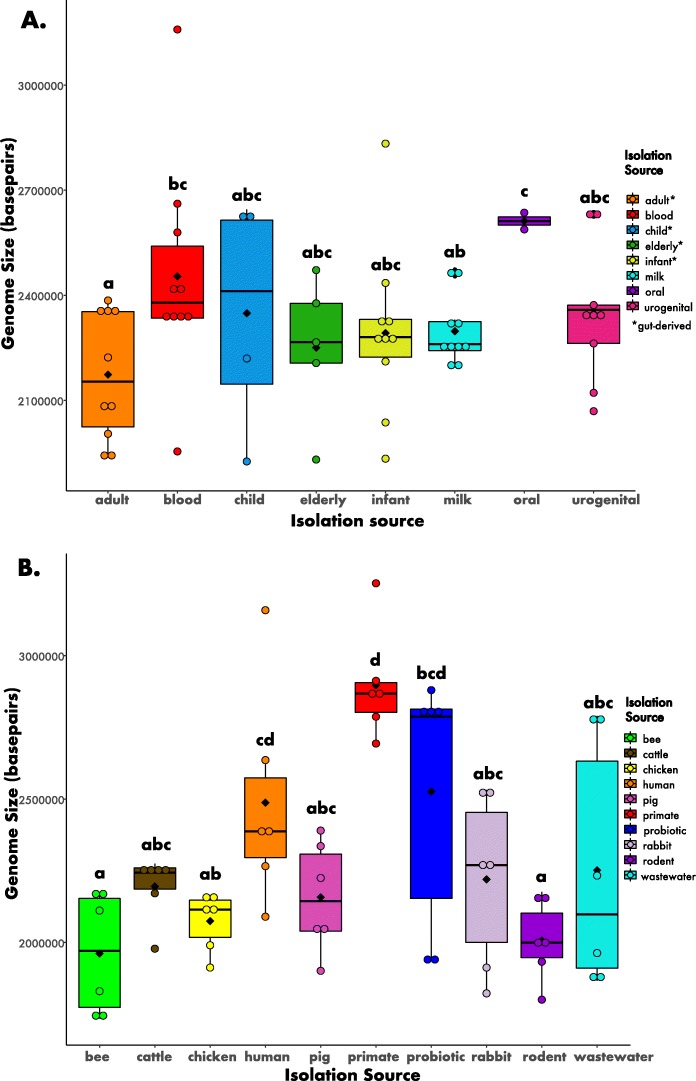
Bifidobacteria genome sizes for strains derived from A) humans and B) multiple hosts and environments. The circles depict the data points, and the black diamonds represent the mean of each boxplot. The letters above each box represent the post hoc comparisons using Dunn’s test where groups sharing a letter are not significantly different. See Methods and Fig. 1 legend for more information about the isolation categories

For the human-derived bifidobacteria strains, the range of genome sizes was ~ 1.9–3.2 Mbp, which falls well within the range of other cultivated human-associated bacteria [41]. Within the human-derived strains, genome size did not differ by the particular human habitat (e.g., urogenital or gut) or between different human stages (e.g., infant or elderly) (Fig. 2a; Kruskal-Wallis H = 10.428, *p* > 0.05, df = 7). Furthermore, the range of genome sizes for bifidobacteria isolated from diverse animal hosts and environments (e.g. primates, bees, wastewater, etc.) was ~ 1.6–3.2 Mbp. These strains differed significantly in genome size, (Fig. 2b; H = 26.244, *p* < 0.01, df = 9). Strains isolated from non-human primates had the highest genome size (2.9 Mbp + 0.19 SD), whereas strains isolated from bees had the lowest genome size (2.0 Mbp + 0.21 SD).

#### Pangenome analysis

**Fig. 3:**
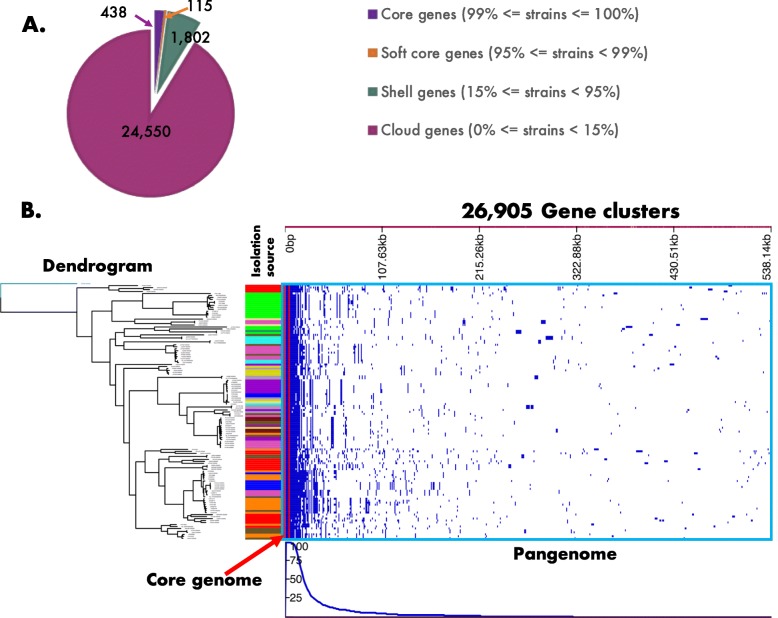
Pan-genome analysis of 129 *Bifidobacterium* strains harbored by multiple hosts. **2A|**The pan-genome of the 129 bifidobacteria strains is summarized in a pie chart showing the core genes (438), the soft genes (115), the shell genes (1802), and the cloud genes (24,550). **2B|**Pan-genome alignment of 129 bifidobacteria strains is depicted by combining the phylogenetic tree inferred by RAxML 8.2.10 and the pan-genome heatmap showing gene presence (royal blue) or absence (white) in each of the strains obtained with Roary 3.11.2. There was a total of 26,905 gene clusters (of orthologous proteins) from which 438 were present in all strains. The line graph at the bottom shows the frequency of genes present within samples. The core-genome and pan-genome are boxed in red and light blue, respectively. The color strip next to the alignment depicts the isolation sources described in Fig. 1

The analysis on 129 bifidobacteria strains revealed that their pangenome is composed of 438 core genes, 115 soft core genes, 1802 shell genes, and 24,550 cloud genes, for a total of 26,905 gene clusters (Fig. 3**)**. This resonates with previous studies with fewer genomes that found this genus to have between 400 and 500 core genes [16,24]. The composition of accessory genes (i.e., their identity and presence absence) excluding the core genome and singletons (~ 6400 genes), was associated with both the bacteria’s isolation source (ANOSIM: R = 0.394, *p* < 0.001), and the phylogeny of the bifidobacteria strains (based on 107 core genes; RELATE test, Spearman’s ρ =0.52, *p* < 0.001).

#### Amino acid biosynthesis analysis

**Fig. 4:**
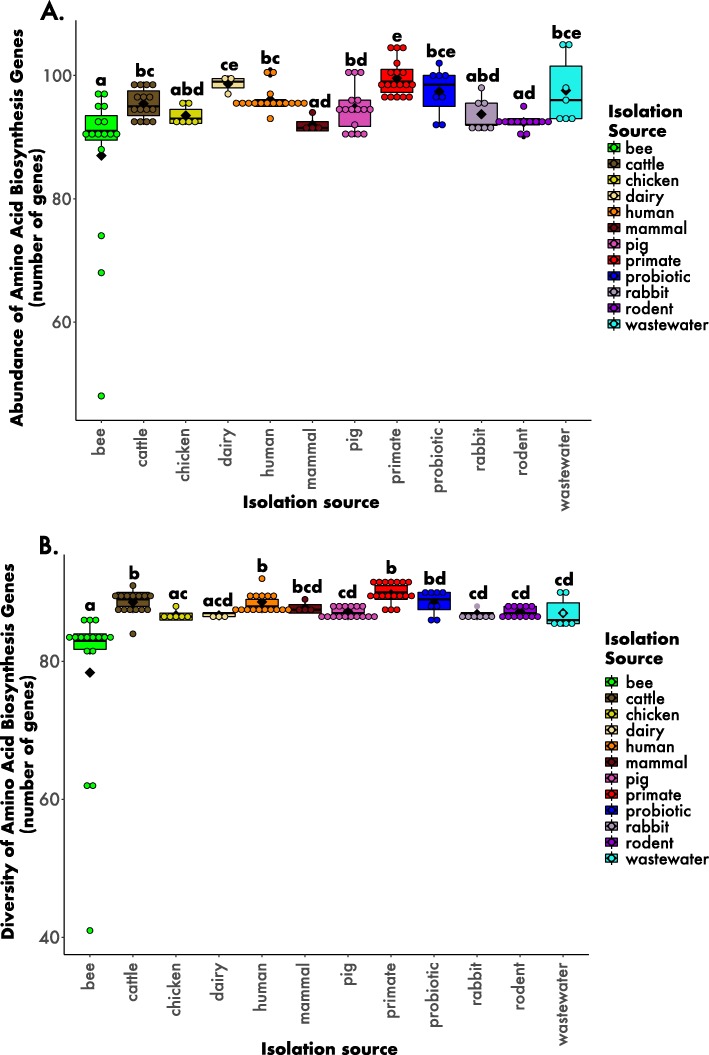
Amino acid biosynthesis gene A) abundances and B) diversity among different hosts and environments. Abundance refers to the number of total gene count and diversity refers to the number of different genes found. The circles depict the data points and the black diamonds represent the mean of each boxplot. The letters above each box represent the post hoc comparisons using Dunn’s test where groups sharing a letter are not significantly different. See Methods and Fig. 1 legend for more information about the isolation categories

Beyond general genomic characteristics, we investigated how a variety of specific traits, such as amino acid biosynthesis genes varied among the strains. There was a significant difference in abundance (number of genes) of amino acid biosynthesis genes between different animal hosts and environments (Fig. 4a; H = 62.216, *p* < 0.001, df = 11) (post hoc Dunn’s test). For instance, bees showed the lowest abundance of amino acid biosynthesis genes (87 genes + 13 SD) while non-human primates showed the highest number (100 genes + 2.9 SD) (Fig. 4a).

Furthermore, the diversity (number of different genes) of amino acid biosynthesis genes also differed among hosts and environments (Fig. 4b; H = 76.594, p < 0.001, df = 11) (post hoc Dunn’s test); the bee-derived strains showed the lowest diversity of amino acid biosynthesis genes (78 genes + 12 SD). Strains isolated from the other host categories carried between 86 and 90 genes (Fig. 4b).

#### Carbohydrate-active enzymes (CAZymes)

**Fig. 5:**
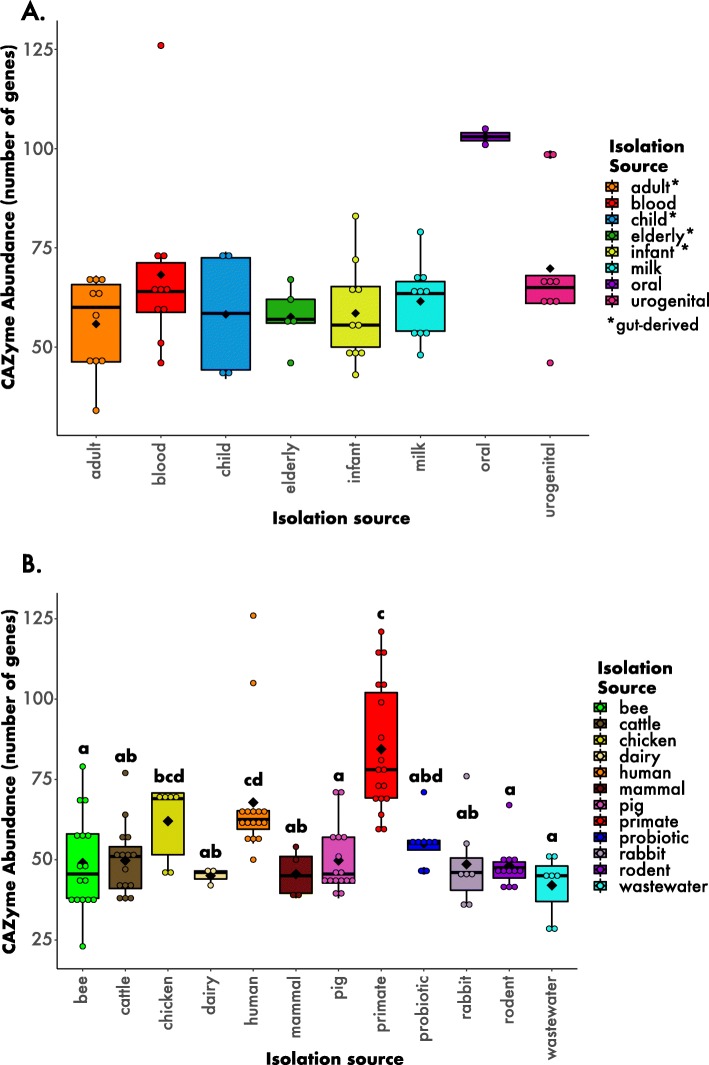
CAZyme abundances for strains isolated from A) humans and B) multiple hosts and environments. The circles depict the data points and the black diamonds represent the mean of each boxplot. The letters above each box represent the post hoc comparisons using Dunn’s test. See Methods and Fig. 1 legend for more information about the isolation categories

Since bifidobacteria are known to be excellent degraders of a variety of carbohydrates, we also searched for CAZymes in their genomes. On the one hand, the abundance of CAZymes among the different human-derived strains did not differ significantly (Fig. 5a; H = 9.6557, *p* > 0.5, df = 7). The oral-derived strains encoded the highest number of CAZymes (103 genes + 2.8 SD), whereas strains derived from adults (gut-derived) encoded the lowest number (55.8 genes + 12 SD). On the other hand, when comparing strains derived across different hosts and environments, we found a significant difference between categories (Fig. 5b; H = 60.9, *p* < 0.001, df = 11). Non-human primates carried more CAZymes than any other host (84 genes + 20 SD), while wastewater exhibited the fewest (42 genes + 10 SD) (Fig. 5).

## Discussion

Studying the diversity of bifidobacteria and their trait associations provides insights into the mechanisms that underlie their assembly within a larger microbial community. Bifidobacteria strains isolated from the same host or environment were non-randomly associated with their phylogenetic relatedness. This pattern is consistent with the hypothesis that bifidobacteria specialize, or at least prefer, particular hosts, in agreement with several other studies [19,24,42]. For example, Lamendella et al. [19] found that bifidobacteria strains from the same host, including those isolated from birds and pigs, tended to cluster by clade. We also observed that all *B. pseudolongum* subsp*. pseudolongum* strains were isolated from pigs as previously noted [43]. Studies have found bee-derived bifidobacteria clustered within relatively deep branches [24,40]. Notably, this was not observed in our study; for instance, some primate-derived strains clustered with more ancient branches than the bee-derived strains. Moreover, rodent and pig isolated strains could be found within several clades. This pattern of imperfect clustering suggests that host-specialization of bifidobacteria has occurred several times within different branches of the genus. In addition, the clades of strains from mixed isolation sources may indicate that many bifidobacteria are not strict specialists but are capable of colonizing non-preferred host types [21].

The bifidobacteria genomes also reveal adaptation to their host environment through genomic signatures like accessory genes and specific gene sets, supporting our second hypothesis. Sun et al. [24] also observed that bifidobacteria isolated from bees, pigs, and humans shared unique sets of genes. However, the correlation we observed between accessory genes and isolation sources was weaker than the association with the phylogeny based on core genes to the whole genus. Thus, it appears that specialization by bifidobacteria to a host species is primarily determined by vertically inherited traits, whereas horizontal gene transfer of traits captured through accessory gene composition plays a secondary role.

More specifically, bifidobacteria strains isolated from different hosts differed in the abundance and diversity of amino acid biosynthesis genes. Notably, bee-derived strains encoded the lowest abundance and diversity of amino acid biosynthesis genes, while non-human primates encoded the highest. Similarly, the bee strains also showed the smallest genome size. Given that previous studies have shown evidence that species isolated from bees, like *B. asteroides*, are more ancestral within the genus *Bifidobacterium* [24, 40], it is possible that bifidobacteria may have coevolved longer with bees than with other hosts. One might speculate a longer coevolutionary history allowed bee-derived bifidobacteria to lose genes by evolving to use amino acids and other nutrients produced by the host or other gut bacteria, similar to the selection for smaller genome sizes observed in obligate bacterial symbionts [30, 34].

Bifidobacteria are also known to degrade a range of carbohydrates ranging from simple to complex molecules, and there was genomic evidence of carbohydrate specialization by bifidobacteria isolated from different hosts. In particular, strains isolated from primates (including humans) carried relatively high abundances of CAZyme encoding genes. This difference could be due to more varied plant diets of primates as well as the complexity and diversity of their milk oligosaccharides [44].

While bifidobacteria strains appear to be adapted to different hosts, there was little evidence that they are adapted to particular habitats and life stages within humans. In particular, we expected that different strains might be adapted to adults or infants, as bifidobacteria composition varies over age [45,46]. Indeed, some subspecies such as *B. longum subps. infantis* are specialized to breakdown HMOs [10]. Perhaps we could not see the pattern at this finer scale due to the limited diversity within each bifidobacteria species in our analysis. However, a recent study also found that strains within just two species, *B. breve* and *B. longum,* isolated from the vagina and gut of humans were indistinguishable based on phylogenetic and genomic trait analyses [22]. Thus, at least for these two habitats, that may be connected by dispersal, there are not specialized strains even when focusing on a finer genetic scale.

The lack of differences in CAZyme abundance among human categories was also surprising. This is contrary to previous studies that have found the highest abundance of CAZymes in gut bacterial communities [8,35,36]. In particular, we expected high numbers of CAZymes from infant strains as some bifidobacteria can degrade HMOs in the babies’ gut allowing the modulation of the immune system and succession of the microbiome in the infants [10,35,47]. A point worth noting is the blood-derived strains, which we suspect are not specialized in their isolation source but instead are transient. Indeed, the strain classified as *B. scardovii* JCM 12489^T^ = DSM 13734^T^ (accession number AP012331) has been reported to have one of the largest genomes consisting of 3,158,347 bp with no plasmids and with the largest number of glycosyl hydrolase genes [48].

Our conclusions are limited by data issues inherent to the reanalysis of publicly available genomes that could be addressed in future research. First, the sampling among host animals is quite uneven, and larger sample sizes among a broader range of hosts would strengthen the results. Second, signals of host or habitat adaptation will be stronger at a higher genetic resolution (i.e. within bifidobacteria species), and thus there is a need for deeper sampling of strains to resolve finer-scale adaptation. Related to this, we had to exclude many human-derived genomes that were not accompanied by information about the specific isolation site and age stage of the host. Lastly, it is unclear whether some of the observed patterns might have been influenced by different isolation methods, which likely varied across different studies.

## Conclusion

This comparative genomic analysis reveals that bifidobacteria are adapted to their hosts. This adaptation is reflected in the evolutionary history of the shared core genome as well as their accessory gene composition and specific gene sets. At the same time, there is little evidence within the genus for specialization on particular human habitats or stages, which may be due to sampling limitations or a higher degree of bacterial dispersal within humans than appreciated. In sum, the assembly of bifidobacteria in their habitats appears to be determined by a mix of ecological (host filtering) and evolutionary (host adaptation) forces [49]. Bifidobacteria thus offers a model to study these processes in animal microbiomes.

## Methods

### Genome sequences and annotation

Genome sequences of all *Bifidobacterium* strains were downloaded from the Pathosystems Resource Integration Center (PATRIC) and the National Center for Biotechnology Information (NCBI) databases on March 14th, 2018 (*n* = 497). Duplicate or wrongly assigned sequences were removed from further analysis. We identified the hosts for each of the strains by searching the PATRIC and NCBI databases or associated publications (*n* = 446) (Additional file 1). Based on the concatenation of 107 core genes (see phylogenetic analysis below for details), we removed sequences with many gaps in the core genes from further analysis (*n* = 400). The vast majority of the strains in the databases were derived from human hosts followed by primates, cattle, pigs and bees. For strains isolated from humans (*n* = 271), we assigned each strain to the most specific category possible, acknowledging that some categories are subsets of other categories: infant (*n* = 117), adult (*n* = 20), human blood (*n* = 13), human milk (*n* = 10), urogenital (*n* = 9), elderly (*n* = 5), child (n = 4), probiotic (*n* = 3), oral (n = 2), human unspecified (*n* = 88). Child refers to 2–6 years old while infant usually refers to children anywhere from birth to 1 year old (or reported as infant in their respecting studies). A subset of 60 human-derived strains from diverse environments were selected based on their unique sequences, descriptive isolation source, and species diversity as to include a variety of *Bifidobacterium* species. Furthermore, we aimed to keep the number of genomes per isolation source/habitat roughly equal (~ 10) whenever possible. When we were borderline between two genomes with the same isolation source and species, we chose the genome that had a “completed” status or a publication to back up the sequence. These 60 isolates were used for genomic comparisons (Additional file 2).

To compare strains among hosts, we focused on a subset of 129 bifidobacteria strains. These strains included the majority of the non-human bifidobacteria, in addition to a subset of human strains from adult and infant feces (*n* = 13), blood (n = 1), vagina (n = 1), and mouth (n = 1). The categories were the following: primate (*n* = 18), human (*n* = 16), cattle (*n* = 15), pig (n = 16), bee (n = 16), rodent (*n* = 12), probiotic (*n* = 8), wastewater (*n* = 7), rabbit (n = 7), chicken (*n* = 6), other mammals (*n* = 4; including giraffe, hippopotamus, llama, and wallaby), dairy products (*n* = 3), soil-plant-associated (n = 1). We recognize that not all the host categories are at the same phylogenetic level (Additional file 3). We selected the human-derived isolates to be included here based on species diversity, isolation source diversity, and only included genomes that were classified as “completed.” Moreover, the non-human isolates were chosen by excluding those that were exactly identical to others based on phylogenetic analysis, and from the identical ones we chose the isolate to include based on species diversity and completion status.

To ensure uniform annotation, we reannotated all the genomes using Prodigal v2.6.3 in Normal Mode to predict Open Reading Frames (ORF) [50]. We then used Prokka v1.13 [51] to annotate the sequences.

### Phylogenetic analysis

Multilocus phylogenetic trees were constructed using the bcgTree pipeline [52] with the protein fasta files (.*faa) derived from Prodigal v2.6.3. Each of the genome sequences was searched for 107 conserved single-copy genes defined by Dupont et al. 2012 [53] using hmmsearch v3.1b2 (Supplementary Table S1 in reference [53]). The extracted genes were then each aligned using muscle v3.8.31 [54] and polished using Gblocks v0.91b [55] by eliminating poorly aligned areas. The 107 genes were then concatenated, and a phylogenetic tree was built using RAxML v8.2.10 with PROTGAMMABLOSUM62 substitution model and 100 rapid Bootstrap searches [56]. We visualized the phylogenetic trees using the iTOL v3 interactive tool [57]. Strains from the *Alloscardovia* genus were used as outgroups for both phylogenetic trees. For the human-derived tree we used an *Alloscardovia omnicolens* (JWAI01000000) isolated from a human and for the multiple-hosts tree we used an *Alloscardovia macacae* from a non-human primate (NEKB01000000).

### Comparative genomic analysis

We next tested whether some of the variation in the traits encoded by bifidobacteria genomes could be explained by the host or environment from which they were isolated. We used the genome size values provided by the PATRIC metadata to compare the genome size among isolates. For human-derived strains we used the same 60 sequences used in the phylogenetic analysis since they were carefully chosen to encompass variable human environments and tried to keep similar sample sizes when possible between categories; however, for the comparison among multiple hosts and environments we used a subset of the 129 strains to keep sample sizes the same for each category (*n* = 6); hence, we did not include isolates from the dairy, mammal, and soil categories since their sample sizes were less than 6 strains (Additional file 3).

The pan-genome and gene ontology of the 129 selected bifidobacteria strains were established with Roary v3.12.0 [58] using the annotated genome assemblies obtained from Prokka v1.13 (.gff files). To account for the relatively high diversity of this genus, we used a 50% sequence identity for the blastp cutoff [59]. The Roary software was able to detect core genes (present in 99–100% of the strains), soft core genes (present in 95–99% of the strains), shell genes (present in 15–95% of the strains), and cloud genes (present in 0–15%). The presence-absence table given by Roary (Additional file 4), depicting the 26,905 gene clusters, was curated by deleting the following genes: core genes present in all 129 strains (minus 352 = total: 26,553), singletons (minus 10,967 = total: 15,586), genes with an average sequence per isolate higher than 1, due to splitting errors (minus 189 = total: 15,397), and genes with hypothetical annotation with no identifiable gene name (minus 9000 = total: 6397). The final table containing 6397 accessory genes was converted into a matrix (Additional file 5**)** for further comparisons between core genes and phylogenetic distance against accessory gene composition. We used Phandango [60] to construct the pan-genome alignment by incorporating the RAxML inferred tree and the presence-absence table given by Roary.

To assess the abundance (number of genes) and diversity (number of different genes) of amino acid biosynthesis genes, the automatic annotation server Ghostkoala was used to obtain gene function assignments based on the KEGG Orthology [61] (Additional file 2, Additional file 3 and Additional file 6). To identify the CAZymes encoded in each genome, we used the dbCAN2 meta server based on the CAZy database updated on July 13th, 2018 [62,63]. The input files for the webserver were protein fasta files (.*faa) derived from Prodigal v2.6.3. This server has the option to utilize three tools to predict CAZymes: i) HMMER search against the dbCAN HMM (hidden Markov model) database; ii) DIAMOND search against pre-annotated CAZyme sequence database; iii) Hotpep search against the CAZyme short peptide database. We used all three tools at the default parsing thresholds and only considered the CAZymes found by all three tools (Additional file 2 and Additional file 3).

### Statistical analyses

We used ANOSIM in PRIMER-6 Software [64] to test whether the isolation source categories were associated with phylogenetic relatedness and accessory genes of the bifidobacteria strains. To test for a correlation between the similarity in accessory and core gene content, we used the RELATE test in PRIMER-6, which is a comparative (Mantel-type) test on similarity matrices [64]. We used the Tree and reticulogram REConstruction (T-REX) web server [65] to create the distance matrices used in the ANOSIM and RELATE tests using the Netwick phylogenetic tree from RAxML. We assessed normality of data using Shapiro-Wilk normality test and its variance with Levene’s test incorporated in RStudio version 1.1.453. To account for the non-normal data and non-equal sample sizes, we used the Kruskal-Wallis (with a calculated significance level of *p* > 0.05) and Dunn’s post hoc tests (RStudio version 1.1.453) to compare genome size, amino acid biosynthesis genes, and CAZymes between the different strains belonging to varying hosts and environments. To construct heatmaps and boxplots, RStudio version 1.1.453 (http://www.rstudio.com/) was implemented and to help with the optimization of the images created, Adobe® Acrobat® Pro 2017 was used.

## Supplementary information

**Additional file 1.** List of all the 446 bifidobacteria isolates and their isolation sources.

**Additional file 2.** List of the 60 bifidobacteria human-derived strains included in the analysis.

**Additional file 3.** List of the 129 bifidobacteria strains across all environments included in the analysis.

**Additional file 4.** Presence-absence table given by Roary.

**Additional file 5.** Accessory gene presence-absence matrix.

**Additional file 6.** KO number assignments for amino acid biosynthesis genes.

## Data Availability

All data generated or analyzed during this study are included in this published article and its additional information files.

## Abbreviations

ANOSIM: Analysis of similarities
CAZymes: Carbohydrate-active enzymes
HGT: Horizontal gene transfer
HMOs: Human milk oligosaccharides
Mbp: Megabasepairs
NCBI: National Center for Biotechnology Information
PATRIC: Pathosystems Resource Integration Center
SCFAs: Short-chain fatty acids
SD: Standard deviation
